# Is It Oscar-Worthy? Children’s Metarepresentational Understanding of Acting

**DOI:** 10.1371/journal.pone.0119604

**Published:** 2015-03-11

**Authors:** Thalia R. Goldstein, Paul Bloom

**Affiliations:** 1 Yale University, Department of Psychology, PO Box 208205, New Haven, CT 06902, United States of America; 2 Pace University, Department of Psychology, 41 Park Row, 13^th^ Floor, New York, NY 10038, United States of America; Boston College, UNITED STATES

## Abstract

Although it is an essential aspect of one of the most common forms of entertainment, psychologists know almost nothing about how children understand the act of portraying a character in a realistic manner—realistic acting. Do children possess the sort of meta-theory of acting that adults possess? In two studies we find that, unlike adults, children between the ages of 3–5 do not think that a realistic actor is better at portraying a characteristic than a nonrealistic actor, nor do they prefer one to the other. As they develop, they come to understand that realistic acting is different from nonrealistic acting, but unlike adults, children think that a nonrealistic, pretense-like portrayal is more difficult to achieve than a realistic representation of an emotional or physical state. These findings show that children’s metarepresentational understanding of acting is relatively immature at age 5, and that their understanding of this specific domain of pretense lags behind their understanding of pretense in general.

## Introduction

Adults enjoy realistic acting. Indeed, watching others act—in television shows, in movies, and in theatre—is one of the main pleasures of modern life [1–3]. Adults also have a common-sense theory of how acting works. They appreciate that while behaviorally realistic acting may be as close to a representation of reality as possible—and, indeed, a convincing match with reality is valued in modern Western acting—it is nonetheless not real. This understanding of the non-reality of acting is central to its appeal: An audience member might be transfixed at someone portraying Hamlet agonizing whether or not to kill himself, but watching someone actually deliberate suicide would not provide the same pleasure (see [2] for discussion).

Our judgments about the praiseworthiness of acting are based in part on our judgments about how close an actor’s portrayal is to reality, how truthful it seems. Realism in the fictional world of realistic television, movies, and plays has many facets—the language, plot, scenery and costumes all play a role. However, we hypothesize that the most important facet of realism in acting is its behavioral cues. The more behaviorally realistic acting is, the better we judge it to be. Indeed, in good acting, there are no behavioral cues to the fact that what the actor is doing is fictional.

In contrast, unrealistic acting is often laughably bad, particularly when it involves over-exaggerated emotional expression or physical movement. The difference between these two types of acting is how close they are to everyday behavior. Behaviorally realistic acting attempts to be as close to everyday behavior as possible, such that if actors were seen engaging in these behaviors on the street, they would not stick out as performative. Emotional expressions are natural, and actors say their lines in a conversational tone with conventional body movement.

To put it differently, nonrealistic acting looks as though the actor is obviously “faking it”. This includes both behaviors that professional actors would think of as overacting and what developmental psychologists have found as “cues to pretense” [4]. This type of acting involves over pronounced facial expressions (e.g. much higher eyebrows and wider-opened eyes when portraying fear than would typically be seen in real contexts or in realistic portrayals of fear), large arm or torso movements that are particularly obvious (e.g. throwing ones’ arms up into the air suddenly and flapping them when portraying surprised), smiles and eye contact with the audience or camera even when it is not appropriate for the state being portrayed (e.g. not only during happiness, but also during tiredness), and exaggerated vocal variation (e.g. close to “motherese” type tones) while speaking. For professionals, this over-exaggeration is thought of as melodramatic; modern Western acting theory and training is based instead on bringing truthfulness to all portrayed situations [5–6].

This focus on behavioral realism in acting is actually a relatively new development. Before the past 100 years, acting styles were closer to oration or even interpretive dance [7]. Even so, the goal of actors has always been to present the truth of life onstage, although theories about the best way to present this truth has changed. Western acting training in particular is focused on actors performing “truthfully” as their characters [8]. In most acting seen today, whether in musical theatre, farce, or movies taking place in outer space, adult audiences respond positively to realistic behavior, with critics complaining and scholars discussing how unrealistic “bad” acting pulls audiences out of being emotionally engaged with the narrative [9]. This type of realistic acting may not be universal or natural, but it is the most prevalent and awarded form of acting seen today. We should note that when we say that realistic acting is appreciated as difficult and rewarded as such, we are not necessarily saying that it is seen as always the most difficult. After all, outlandish physical slapstick might be seen as harder than run-of-the-mill realism. Musical theatre in which actors have to sing, dance and make it all seem normal may also be seen as harder to perform well than straight plays. Our point is simply that realism is still seen as critical and difficult.

Behavioral realism in acting comes with a cognitive price for the audience: the more realistic acting is, the harder it becomes to separate it from reality. The representational difficulties of separating actor from character are even built into the way we talk about acting. Actors “inhabit” roles, or “take on” parts. This is distinguished from pretense—it would be strange, for instance, to hear Daniel Craig described as “pretending” to be James Bond, but quite natural to say that Daniel Craig *is* James Bond. Indeed, there is anecdotal evidence that adults sometimes confuse the representational nature of high-quality realistic acting by attributing the properties of a character to the actor. For instance, Hugh Laurie has mentioned in several interviews that he is often asked for medical advice due to playing Dr. Gregory House for nine years on television. Leonard Nimoy, who played the Vulcan Mr. Spock on Star Trek, was subsequently plagued throughout this career with people who believed that Nimoy himself was hyper-rational and emotionless, so much so that he titled his autobiography *I Am Not Spock* [10] (although he has since recanted and proclaimed that he is, indeed, Spock [11]). Advertisers used to exploit this confusion by using actors that played doctors to advertise medicines. A commercial for cough medicine in the mid-1980’s actually used an actor from the show *General Hospital*, who said the line “I’m not a doctor, but I play one on TV” to explain to the viewer why they should buy the product.

How does the capacity to appreciate realistic acting develop? The average American preschool-aged child spends between 90 minutes and two hours watching television and DVDs per day [12]. Much of this exposure includes shows that contain acting [13]. Despite its prevalence in their everyday lives, children’s understanding of realistic acting is an unexplored domain. We do not know if children can discern the importance of realism in the representation of enacted characteristics, or whether children can tell what makes “good” acting. We do not know whether they understand realistic acting at all—no work that we know of has looked at children’s reactions to and judgments of realistic portrayals of fictional humans.

The developing understanding and appreciation of realistic acting in children is interesting for several reasons. For one thing, realistic acting can serve as an unusual and particularly challenging case study of children’s developing meta-representational understanding. A mature understanding of realistic acting engages multiple representations—the person you are watching is both the actor and the character that the actor is representing. The actor has his or her physical, emotional, and personality states and traits, and the character/portrayal has his or her emotional, physical and personality states and traits. Previous research has shown that for emotional states particularly, children typically have problems identifying that a person can feel one way internally but express a different emotion until after five years old [14–15]. Other research from our lab shows that preschool children misunderstand representation of enacted traits; until they are about five years of age, and unlike adults, they almost always believe that actors experience the mental and physical states of the characters they portray. This may imply that children do not think of acting a representational at all: they may instead think that actors, regardless of type of portrayal, are simply experiencing the characteristic they are acting. They may believe that acting is the same as real life, which is why it looks like real life [16]. If this is the case, it would reveal a dramatic difference between the everyday experience of children and of adults.

Studying the domain of realistic acting is interesting as well in how it contrasts with other representational challenges. For instance, even young children show a relatively mature understanding of representation in pretend play. Just as with realistic acting, children engaging in pretend play must hold multiple representations in mind as they switch from the real world into the pretend one [17–18]. But pretense poses little to no challenge for adults and most young children. As young as eighteen months old, children understand pretend play. They understand that an object can be both itself and a pretend object, and by three years old rarely, if ever, make a mistake about an object’s *actual* versus *represented* properties [19–21]. At this age, children understand that objects used in pretend are representational (e.g., a pencil can represent a rocket ship [22–23]), and that the same real object can represent different pretend objects in different scenarios [24]. By three years old, children can also tell the difference between someone who is trying to complete an action, and someone who is pretending to complete an action [25] and can distinguish real and pretense acts even without cues such as the presence or absence of a target object [21]. Preschool children themselves begin to take on pretend roles at this age [26], although this role play is not thought of as realistic.

Why would pretense be so much easier than realistic acting? This is likely due to the abundant behavioral cues in pretend play that distinguish it from real-world actions: these cues make its representational nature obvious. These cues are necessary in part because pretense is embedded in the everyday activities of children. Children and their caretakers often move from real interactions to pretend ones, and therefore must demarcate the pretend world with behavioral and vocal cues. These cues include: extensive interpersonal eye contact, non-context appropriate smiling, high levels of vocal variation, and over-the-top physical actions [4, 27–28].

Realistic acting, a close representation of the real world, does not contain these cues. If someone were to ask how children distinguish the behavior manifested in realistic acting from actual real world behavior, the answer is that—based on the solely on the behavior—they cannot; the whole point of realistic acting is that, when done well, it is indistinguishable. If someone were unfamiliar with the external cues to acting, such as the framing of television, movie screens, or proscenium arches, or the genre cues that distinguish acting from realistic filming (such as music and camera angles), he or she would not be able to tell the difference between realistic acting and reality. When not given the appropriate framing cues, even adults can confuse realistic acting and reality, a popular example being the famous first broadcast of War of the Worlds, which was only voice acting.

(Note that this is different from the confusion mentioned above, where adults struggle with the distinction between Leonard Nimoy and Spock, or Hugh Laurie and an actual doctor—in those cases, they are consciously aware that realistic acting is taking place, but fail to inhibit certain generalizations that would hold if the characters were real.) Children must learn the conventions of fictional framing—title cards and opening theme songs, laugh tracks and montages—in order to understand that television and film are fiction in the first place [29].

This understanding of realistic acting is intimately connected to the pleasure that we get from it. For adults at least, one possible source of this pleasure is the enjoyment we derive from an appreciation of the dual representation implicit in acting: the actor versus the character. We give awards and large sums of money to those who can create realistic portrayals of emotional and physical states, and do it well. Much as we enjoy watching talented individuals run races, kick soccer balls, and sing arias, we enjoy watching people complete the task of portraying human psychologies on a stage or screen [3].

In this regard, children’s appreciation of realistic acting provides a window into their understanding. That is, while children may not be able to explicitly outline the complex logic of realistic acting, they can tell us what they like. If children agree with adults that realistic acting is better than nonrealistic acting, that means that, among other things, they understand that acting is about generating an impression that is indistinguishable from reality. However, if children believe that nonrealistic acting is better than realistic acting, that means they may instead believe that behavioral cues that makes something as unreal are important for the quality of a performance. They might either be incapable of understanding that one can behave in a realistic way while acting, or they might think that someone who acts realistically is doing it wrong.

In two studies, we compare children’s understanding of realistic acting with their understanding of overt and exaggerated nonrealistic acting—behaviorally similar to what they engage in during everyday pretend play [4, 20, 30–31]. We do not provide any other cues: all clothing and scenery are the same, and there is no plot, only enacted expression of emotional and physical states. We investigate how children think about what constitutes acting using simple forced choice questions. We compare examples of realistic representational acting with examples of overt, nonrealistic, and overacted, but still representational, acting to see if children’s understanding of acting is the same as adults’.

## Study 1

In Study 1, to investigate what children think is good acting, we introduced children to an actor who wanted to be on “television and movies like some shows that you watch.” We then showed them two versions of the same actor enacting the same characteristic: once in an overblown manner, closer to pretense, and once in a realistically acted manner, asking children to decide which they thought was the best.

Children could respond in one of three ways: 1) If children choose realistic actors as “better”, this would imply that children have an adult-like understanding of the importance of realism in acting. 2) If children choose the nonrealistic actors as “better”, this would imply that children do not appreciate that realism in the representation of an enacted characteristic is important, and/or may instead be choosing based on shows that they themselves watch, or cues they find in their own pretense. 3) If children do not have a preference either way, they may not understand that realistic and nonrealistic portrayals are somehow different. Alternatively, they may not have a preference because they may both understand that behavioral realism is acting is important, and think that nonrealism is important because of what they see on their own television programs, and therefore answer randomly.

To ensure the target actors really did show differences between realistic acting and nonrealistic acting, we also had adults rate which acting style they believed was “better” for television and movies.

### Method

#### Participants

Participants included 17 3-year-olds (38 to 45 months, *M* = 42 months; 11 female), 16 4-year-olds (48 to 59 months, *M* = 53 months; 7 female), 13 5-year-olds (60 to 69 months, *M* = 63 months; 8 female), and 10 adults (19 to 66 years, *M* = 39 years, 1 months; 5 female). Ethnic information was not collected, but most participants were representative of the predominantly Caucasian population from which they came. Child participants were recruited from a database of families who had agreed to participate in developmental research or were students at a preschool that agreed to participate in psychological research. Adult participants were either working in a psychology laboratory and had no knowledge of the study, or were recruited while walking across campus and participated in exchange for candy.

#### Materials and Procedure

All research reported in this manuscript was approved by the Yale University Institutional Review Board (IRB). Informed consent was obtained in writing from all participants or their parents, and verbal assent was obtained from all children during the course of the study. Two videos (one realistic acting, one nonrealistic acting) were presented side-by-side on the same screen in a Power Point presentation. Children were told “*Now we’re going to play a game, where I show you some videos of people acting. Do you know what acting is*? (Child Response)”. *Acting is when someone plays different characters in movies or plays. In this movie, people are acting. What is your favorite movie*? This discussion lasted a few minutes, and the experimenter made sure that the child did not only talk about cartoons, but also live action, enacted characters from TV shows and movies. Then children were shown two exemplar videos of the same actor portraying two different characters, from a television movie aimed at children, a modern retelling of “The Prince and the Pauper” [32]. They were told “*Do you know who this is*? (Experimenter shows picture of actor) *This is someone who is acting. See, in this video, he is acting as a character*. (Experimenter shows video) *And here he is acting as another character*. (Experimenter shows second video) *So, do you know what acting is*? (Child responds).

Children then watched our target videos. They first watched one video, then the other, and then answered our questions. Type of portrayal was counterbalanced by side across trials, as was the order in which the conditions were presented. There were eight sets of characteristics: happy, sad, scared, surprised, tired, strength, food preference, and hurt. For each set of characteristics, the same actor portrayed a single characteristic (i.e. happy, sad, tired, or hurt), but in two different ways: in one condition, the actor acted realistically and in the other, the same actor acted in an overblown nonrealistic manner. In both conditions, the target walked across the screen, stopped at a desk, had an emotional or physical reaction, and then continued off screen. In the realistically acted condition, the responses were as realistic as possible, while in the nonrealistic acting condition, the targets acted in an overblown and obviously fake manner: they made extensive eye contact with the camera, smiled even when portraying a negative emotion or physical action, and had higher levels of vocal expression and variation than the realistic acting, all signals of pretend as found in previous research [4, 27–28] and mentioned earlier. Videos were between 10–15 seconds long. The action, script, background and actors’ clothing were identical for both conditions; it was the vocal intonation, facial expressions, and physical reactions that differed. Table 1 lists the action and script used for each characteristic.

**Table 1:** Characteristic type, wording of scenario.

Characteristic	Action	Script
Happy	Receiving a present	“Yay! I love presents! This is so exciting!”
Sad	Dropping a glass	“Oh no! I dropped the glass. I shouldn’t have done that. Oh no.”
Scared	Seeing a mouse	“Ahh! Look at that mouse! Ahh!”
Surprised	Receiving and opening a letter	“Oh my gosh, that’s amazing, I didn’t know that.”
Tired	Doing two jumping jacks	“Whew, I am so tired. I don’t think I can do another jumping jack, I am so tired.”
Strength	Attempting to pick up a small object	“Ooh, wow that is really heavy. I don’t think I could lift it by myself.”
Hurt	Pain in the knee	“Ow, my knee hurts, that is really painful.”
Food Preference	Choosing raisins over nuts	“Ooh, raisins, they’re my favorite. Bleh, I don’t like nuts.”

After watching both videos, children were asked “Which one is better? Which one is the best?” While answering, children were looking at a screen shot, frozen, from the same moment of the end of each video while making their choices. For each choice, children were given a score of “0” for choosing “realistic acting” and “1” for choosing “nonrealistic acting”. Therefore, children could score between 0 (choosing all realistic acting) and 8 (choosing all nonrealistic acting). Examples of “realistic acting” and “nonrealistic acting” videos can be found at osf.io/ms9wu. All data for this study are freely available through the Open Science Framework and can be found at https://osf.io/kn56y/.

### Results

**Fig 1:**
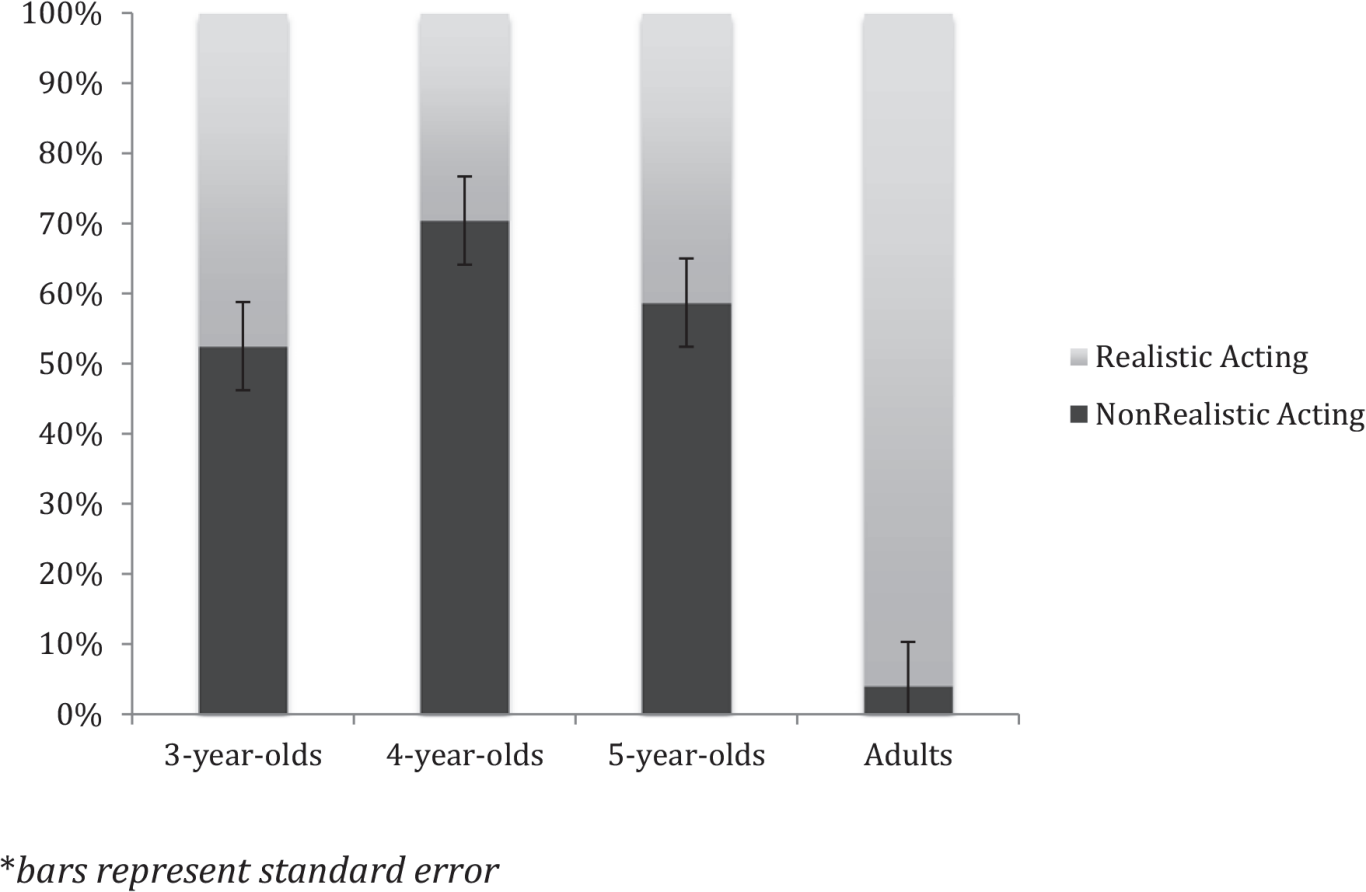
Choice of “better” acting type, by age.

To investigate whether there was a developmental trend in choice of “better” acting, we conducted a one-way ANOVA with age as the between subject variable. Fig. 1 presents the results by age.

There was a main effect of age, *F* (3, 52) = 15.41, *p* <.001, *d* = 2.13. Tukey’s post-hoc tests showed that adults were much more likely to choose the realistic actor as “better” than the 3- 4- or 5-year-olds (all *p*s < .001), but there were no significant differences between the 3- 4- and 5-year-olds. We then reran the ANOVA without the adults and there was no significant effect of age.

We then examined the results at each age by conducting *t*-tests to see if participants responded that the realistic actor was “better” differently from chance, understanding that acting should be realistic in its behavioral cues. Adults were significantly more likely to say that the realistic actor was “better”, *t* (9) = 8.40, *p* < .001, *d* = 5.6. Both the 3- and 5-year-olds responded at chance. However, 4-year-olds were more likely to say that the nonrealistic actor was “better”, *t* (15) = 3.77, *p* = .002, *d* = 1.94.

### Discussion

In sum, while adults thought the realistic actor was better, children did not. They either showed no preference (3- and 5-year-old children), or thought the over-the-top, nonrealistic actor was better, more like the acting they saw on movies and TV (4-year-old children).

There are at least two possible explanations for the developmental difference between children and adults. First, it is possible that the 3- and 5-year-old children cannot tell the difference between nonrealistic and realistic acting. They may think that both are valid representations of an emotional or physical state and not be able to distinguish between the two despite the perceptual differences in the behavior. So, these children may have an understanding of the *representational* nature of both realistic and nonrealistic acting, they may not understand the *metarepresentational* properties of acting: that a nonrealistic representation is not as good as a realistic representation. However, if this is the reason for the 3- and 5- year olds’ responses, this means that only the four-year-olds can tell the difference between realistic and nonrealistic acting—that only at this intermediate age could children understand the metarepresentational nature of acting (but understand it incorrectly, believing a nonrealistic portrayal is better). We find this explanation unlikely, given the pattern of age differences found in our study. Clarification of this issue was one motivation for Study 2.

Secondly, children were introduced to the study by talking about movies and television they watch regularly, and then asked about the movies and television shows they watch regularly. A lot of children’s television (e.g. *Blue’s Clues* and *Yo Gabba Gabba*) involves unrealistic and exaggerated behavior. The four-year-olds in our study could have been responding in reference to the type of acting they see on their television shows, although the three- and five-year-olds were not. Although we did not measure children’s exposure to television shows in this study, as mentioned above, preschool children in the United States watch on average 90 minutes to two hours of television and DVDs per day [12]. It is therefore likely that the children in our study had at least some exposure to the non-realistic and more pretense-like acting of characters specifically made for children’s television.

This would explain the 4-year-olds’ responses. But surprisingly, 3- and 5-year-old children did not choose either option as better. If these children do pay close attention to the type of acting they saw on their favorite shows, it is likely than children would have thought the nonrealistic actor was a “better” actor, rather than who adults thought was the better actor (i.e. the realistic actor). This lack of preference could support the theory that 3- and 5-year-old children did not take acting style into account when deciding their preferences for actors in the same way as adults.

We admit, then, that neither explanation fully captures the differences in responding between the 4-year-olds and the 3- and 5-year-olds. It may be that the three- and five-year-olds look as though they responded in the same way, but do so for different reasons. This is an admittedly post-hoc explanation, but it may be that 3-year-olds were confused about the representational nature of the two types of acting and therefore equated them; four-year-olds preferred the nonrealistic acting because this is what they see on television and in their own pretend and do not understand the importance of realism in acting, and five year olds are beginning to understand the importance of realism in acting, but were confused or still have a preference for nonrealism given their pretense and television exposure, and therefore appeared to answer randomly. However, it should also be noted that while the four-year-olds did respond differently from chance, their pattern of responding was not significantly different from the pattern of responding from the 3- and 5- year-olds as found by an ANOVA. As our primary goal is to investigate children’s understanding of the differences between the two types of enactment, we attempted to focus on this question in Study 2.

Finally, it is possible that children did not understand the concept of acting as we explained it in the instructions. Children may have conceptualized the video clips as simply real expressions of real emotions, not thinking of them as representational at all.

Study 2 attempts to clarify these alternatives, expand our previous findings, and provide children with a clearer way to distinguish between realistic acting and nonrealistic acting. In Study 2, we provided children with an example of what we meant by realistic acting, rather than only talking with them about the movies and television they experience in their daily lives. We showed children the same portrayals of characteristics as in Study 1. In Study 1, it may have been that children also interpreted our target question differently than adults. They may have interpreted our question of “which is better” (as in, which is the better actor) to mean “which do you like better”, and therefore answered with their preference rather than with a judgment. We clarify this possibility in Study 2 by giving children a chance to distinguish between what kind of acting they prefer and what kind of acting they think of as more like the realistic acting adults value. We also ask which kind of acting they consider to be more difficult to explore whether children understand that part of an appreciation of acting’s representative nature is an appreciation of the difficulty of creating that representation. In this way, we can clarify the reasons behind the similarities in three- and five-year-old children. These questions help to illuminate the results from Study 1 and see if there are aspects of realistic acting, such as its difficulty, that children between the ages of three and five represent and understand in the same way as adults.

## Study 2

In Study 2, we introduced the children to an actor who “played different characters on movies and television” and showed them a sample of realistic acting. We then showed them two versions of other actors either realistically or nonrealistically enacting the same characteristic—and asked children to pick which one was more similar to the sample of realistic acting, which one they liked better, and which one they believed was harder to do.

There are again three possibilities. 1) If children have a mature understanding of realistic acting and they understand the centrality of realistic behavioral cues in acting, they should respond that our realistic acting videos are the same as the realistic acting exemplar, prefer realistic acting, and believe realistic acting is more difficult to achieve than nonrealistic acting. 2) If children do not understand the representational goals of acting and instead believe that “bigger is better” when it comes to portrayals, or that realistic behavioral cues are not actually representational, only over-the-top portrayals are, they may respond that our nonrealistic acting videos are the same as the realistic acting exemplar, prefer nonrealistic acting, and believe nonrealistic acting is more difficult to achieve. This response pattern may imply that children think of our target realistic acting as the same thing as real behavior—not representational at all and not difficult to perform—and therefore not the same as the exemplar video, which is introduced as a representation. Children’s preferences for nonrealistic acting and belief in the difficulty of nonrealistic acting would then imply that only nonrealistic acting was considered representational, separate from real behavior from our realistic actors. 3) If children equate any type of human representation (realistic or not) they may answer at chance.

We again asked adults, this time using a larger online sample, to rate the videos on the same characteristics as the children as well as on which portrayal type was more typical of what they saw on movies and television.

### Method

#### Participants

Participants included 14 3-year-olds (34 to 47 months, *M* = 40.8 months; 5 female), 15 4-year-olds (48 to 59 months, *M* = 52.7 months; 9 female,), 15 5-year-olds (60 to 72 months, *M* = 63.1 months; 2 female), and 43 adults (18 to 60 years, M = 28 years, 6months; 13 female). Most participants were Caucasian, reflecting the community from which they came. Child participants were recruited from a database of families who had agreed to participate in developmental research or were students at a preschool that agreed to participate in psychological research. Adult participants were either working in a psychology laboratory (10 participants) or were recruited via Amazon’s Mechanical Turk (33 Participants) and had no knowledge of the goals of the study.

#### Materials and Procedure

In the same manner as Study 1, children were first introduced to the concept of acting and asked to talk about their favorite movies and television shows, with the experimenter explaining that on those shows, people were acting. They were then shown a clip from the same television movie as in Study 1 [32], introduced by the experimenter as “*In [child’s favorite] movie, people are acting just like my friend Josh here. Josh is an actor, someone who plays characters on movies and TV. Now we’re going to watch Josh do some acting.”* (Experimenter shows acting scene). In this clip, a child actor is acting in a realistic manner, having a conversation with a security guard about the possibilities of jobs on a movie set. The style was conversational and realistic, and even though it takes place in a television movie aimed at children, the style is such that it could be part of most realistically acted films or television shows. The character Josh asks a question with some hope, and then expresses mild disappointment upon receiving a negative answer. There is no overlap in the physical or emotional state of Josh with the emotional or physical states shown in our target videos. The children were told “This is Josh. Josh is acting. After you watch this, I am going to show you some more people and ask who you think is acting more like Josh.”

Once they watched the sample video, children were shown a series of paired videos, similar to Study 1. Two videos were presented side by side, and both videos were presented before children were asked questions. Videos were again counterbalanced by type. Children saw 6 videos. The *sad* video was not shown due to experimenter error in transfer between computers, and the *food preference* video was not shown due to wanting to keep the number of physical and emotional videos equal in this study.

In each set of videos, two actors again portrayed a single characteristic (i.e. happy, scared, tired, hurt) in two different ways: in one video, the actor acted realistically and in the other, another actor acted in an overblown nonrealistic manner. After watching both videos, children were asked three questions: “Which one was acting more like Josh?” “Which one did you like better?” and “Which one do you think was harder to do?” We asked the questions in this set order and with this wording to orient children’s responses back to the initial target video of realistic acting, and to ensure that children were thinking about realistic acting in their responses. Children again looked at two frozen screen shots from the same moment of each video while making their choices. Children were scored for how often they chose realistic acting rather than nonrealistic acting.

Adults were told that they were participating in a study of acting. They were also shown the sample video and then the target videos and asked the same questions, but without a lengthy introduction about the definition of acting. The Mechanical Turk sample was also asked one additional control question: “Which type of acting was more like what they typically saw on television and movies?” All data for this study are freely available through the Open Science Framework and can be found at https://osf.io/kn56y/.

### Results

To investigate whether adults believed that our videos of realistic acting were more like the type of acting they saw on movies and television than our nonrealistic acting, we ran a one-sample t-test against chance. Adults from our online sample believed that our realistic acting videos were more like the acting they saw on movies and television, *t*(32) = 2.60, *p* = .014.

**Fig 2:**
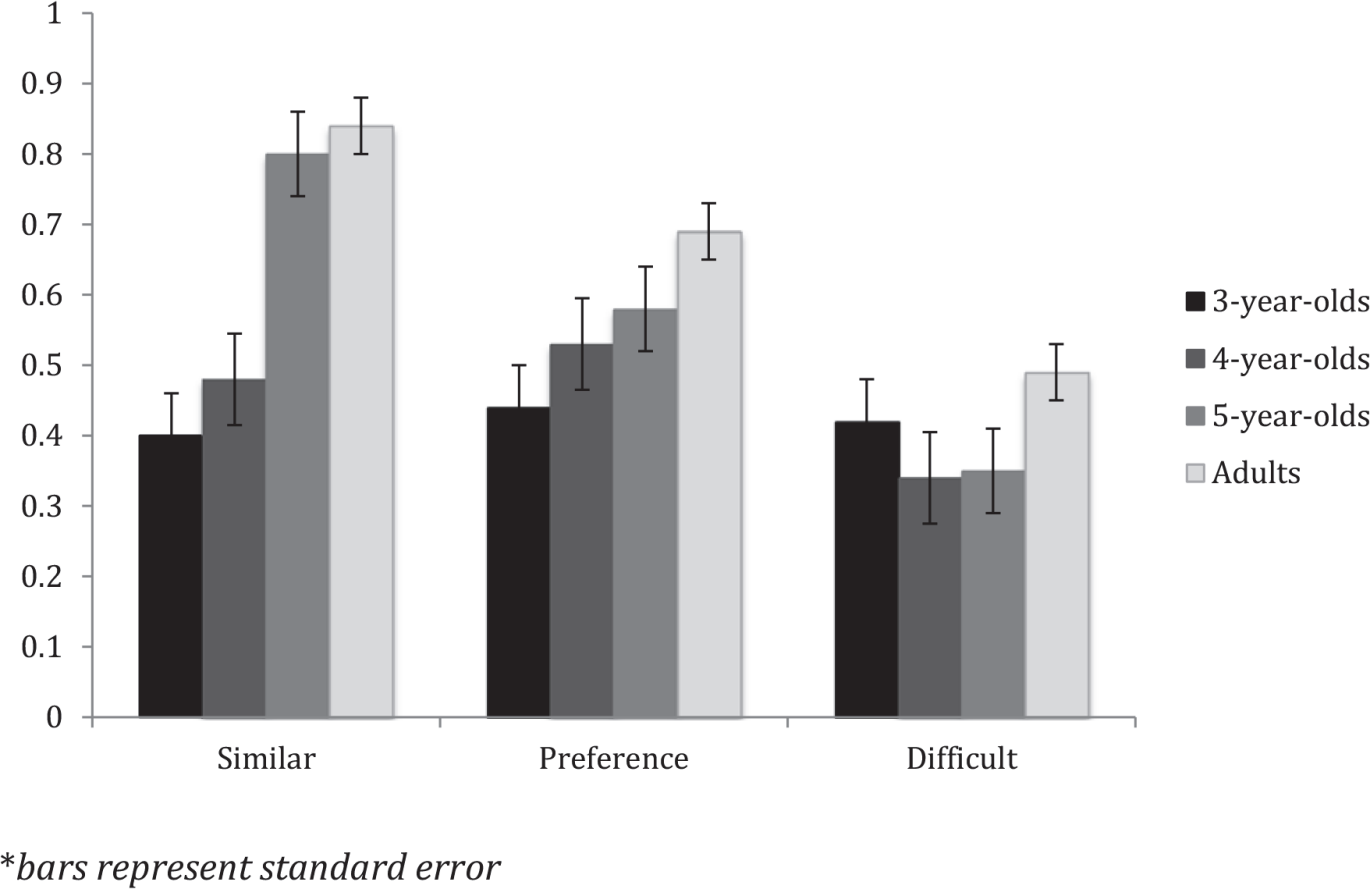
Choice of realistic acting for “similar”, preference, and difficulty by age.

To investigate whether there was a developmental trend in ability to differentiate, preference for, and understood difficulty of realistic compared to nonrealistic acting, we conducted a 4 (age group: 3-year-olds, 4-year-olds, 5-year-olds, Adults) X 3 (question: similarity, preference, difficulty) mixed-model ANOVA. Fig. 2 presents the results by age. Bars indicate percentage of choice of realistic acting for each question.

There was a main effect of question type, *F* (2, 82) = 12.39, *p* < .001, *d* = 1.36 and a main effect of age, *F* (3, 83) = 15.32, *p* < .001, *d* = 1.68, this was qualified by a significant interaction of question type and age, *F* (3, 83) = 4.28, *p* = .007, *d* = 0.46. Follow up ANOVAs showed that there were developmental differences for similarity, *F* (3, 83) = 22.26, *p* < .001, *d* = 2.44, and preference, *F* (3, 83) = 5.26, *p* = .002, *d* = 0.58, but not for difficulty, *F* (3, 83) = 1.43, *p* = .24. Tukey’s posthoc tests showed that adults answered differently than 3-year-olds on the questions of preference (*p* = .002), and differently from 3- and 4- year olds (*p*s < .01), but not 5-year olds, on the question of similarity, but that all four age groups answered similarly on questions of difficulty.

We then re-ran the ANOVA without the adults. There was a significant interaction of question and age *F* (2, 40) = 4.16, *p* = .004, *d* = 0.65, and there was a main effect of question type, *F* (2, 40) = 6.73, *p* = .003, *d* = 1.06, but no main effect of age. Follow-up ANOVAs showed that there was a developmental difference for the question of similarity, *F* (2, 41) = 16.56, *p* < .001, *d* = 2.58. This occurred because 5-year-olds answered significantly differently than both 3- and 4- year olds, *p*s < .001. There was no effect of age on questions of preference or difficulty (all *p*s > .2). While the three questions were asked in the same order, to ensure that choice of the video more like the exemplar was the same as the choice of preferred video, we conducted a bivariate correlation on choice of video for each question. For all children, there was only a moderate correlation between responses to the question about preference and responses to the question about similarity, p = .081. Across question type, for the videos “tired” “surprised” “happy” “hurt” and “strength” overall children’s response was not significantly correlated. For the videos “scared”, responses were moderately correlated, p = .057.

We then conducted *t*-tests to determine if children and adults answered differently from chance for each question. Adults believed that the realistically acted videos were more like the example, *t*(42) = 10.17, *p*<.001, *d* = 3.13, and preferred the realistically acted videos, *t*(42) = 5.75, *p* = .001, *d* = 1.77, and but did not think that the realistic acting was more difficult to do than the nonrealistic acting, *t*(43) = 0.07, *p* = .94.

Three-year-olds answered that the nonrealistic acting targets were significantly more like the realistic acting example, *t* (13) = 2.51, *p* = .02, *d* = 1.39, but did not prefer nor did they think either type of acting was more difficult. Four-year-olds did not differ from chance for which type of acting they preferred or which they thought was more similar to the sample acting. However, four-year-olds answered that the nonrealistic acting was more difficult to do, *t*(15) = 2.51, *p* = .025, *d* = 1.29. Five-year-olds answered that the realistic acting was more like the sample, *t* (14) = 6.874, *p* <.001, *d* = 3.67, and that nonrealistic acting was more difficult than chance, *t* (14) = 2.16 *p* = .048, *d* = 1.15, but did not differ from chance in their preferences.

It is also possible that those participants who did not match our exemplar realistic acting with our target videos of realistic acting answered the preference question differently than those who were able to match the two realistic acting videos, regardless of age. Therefore, we created a new variable, dividing participants into two groups: those who correctly matched on 5 or 6 of the test videos, and those who correctly matched on 4 or fewer videos regardless of age. We then conducted a univariate ANOVA with age and amount of correct matching as predictor variables and preference as the dependent variables. Participants who matched 5 or 6 videos on similarity significantly preferred realistic over nonrealistic acting, *F*(6, 86) = 5.02, *p* = .028, *d* = 1.08. Neither age nor the interaction of age and matching of similarity were significant predictors of preference for realistic acting, *p*s >.27. It should be noted that no 3-year-old children answered 5 or 6 matching questions correctly, and only 2 four year olds did so, while 11 (out of 15) 5-year-olds did so.

### Discussion

The findings suggest that as children develop, they begin to distinguish more clearly between nonrealistic acting and realistic acting. Five-year-olds have mature ability to match realistic acting style of professionals to realistically acted characteristics created for our laboratory, but 3- and 4-year olds have difficulties differentiating between the two, with 3-year-olds equating nonrealistic acting with realistic acting.

One area where there is a difference between children and adults concerns the relative difficulty of realistic acting and nonrealistic acting. As preschool children develop, they think that nonrealistic acting is harder to perform than realistic acting. This is unlike adults, who do not endorse the idea that either type of acting is more difficult. (We had expected that they would find the realistic acting more difficult; the fact that they didn’t may be due to the matched and unnatural nature of our realistic and nonrealistic stimuli). This belief may signify a problem with children’s understanding of realistic acting as both representational of the real world and false. They may believe that the behavioral cues of the actor are so close to reality that they are *not actually representational*, but really happening, and therefore not difficult to portray. In contrast, adults understand that both the realistic and the nonrealistic acting are portrayals, and believe, at least in our videos, that both types of acting are equally difficult.

Although children’s television is nonrealistic in its acting, and certainly cartoons are not realistic, children do not seem to have a preference for one type of acting over another. It may be that if we had asked children instead which type of acting they found more entertaining, not just which one they liked better, but which was more fun, or more interesting, they would have chosen the nonrealistic acting, reflecting the type of acting they see on children’s television.

One additional possibility for these findings is that children believe that “bigger is better and harder to do” across domains, with acting included in their theory. Children may have applied this theory to all questions about acting, thinking that bigger portrayals of emotions or physical states are more difficult to create, and preferable over small, more realistic portrayals. If this is children’s operating theory of not just acting but action in general, it may be that if we presented children with a choice between someone jumping a “big” versus “small” jump, or stringing a set of “big” beads or a set of “small” beads, children would always chose the larger choice.

## General Discussion

In two studies, we found that young children do not have consistent views as to whether a more or less realistic enacted portrayal is better, and they believe a less realistic enacted portrayal is more difficult to achieve. In both regards, children are different from adults. In our studies, adults show a clear understanding of the dissociation between nonrealistic and realistic acting—they believe realistic acting is better and prefer realistic acting. In contrast, our data suggest that preschool children are confused by realistic acting, though they do not prefer nonrealistic, exaggerated acting.

It may be that children are confused by the representational task of acting, that of understanding the difference between realistic behavior and fictional enactment. This is more difficult, we believe, than understanding the obvious behavioral representation of pretense, which preschool children do with ease [17–18, 22–23]. As the behavioral cues of realistic acting are so close to reality, perhaps children do not even consider it representational, in the same way as they do pretense. In most research on children’s understanding of pretense, the use of pretense is either explicitly stated, or the cues to the pretense nature of the world are obvious (e.g. by using blocks to represent other objects, or through the types of behavioral cues discussed earlier). Future work in this area should be careful to ensure the participants fully understand the representational nature of acting, and to explore methods that will help young children understand that what they are watching is a representation, even with its realistic behavioral cues. It may be, however, that even when clarified, children will continue to be confused, as adults sometimes are.

Alternatively, children, unlike adults, may be thinking of all human representation of traits in the same way regardless of the type of perceptual cues that are given. Three-year-olds, in particular seem unable to separate the realistic from the nonrealistic acting, and may in fact think of less realistic acting as being more obviously a “representation” and therefore more closely matched to the sample of acting that we showed them at the beginning of Study 2. In the same way that at three and four years old children are still confused by appearance-reality tasks [33] and tasks that ask children to distinguish between an internal emotion and the expression of that emotion in an attempt to fool someone [14], perhaps children are responding with the belief that an obvious portrayal of a false state (which our nonrealistic acting was) is a better portrayal of that false state than a more realistic portrayal of a false state. This obvious portrayal may help children disentangle an otherwise confusing task: one that involves realistic perceptual cues, but a framing of representation. In this way, change in children’s ability to separate layers in acting could be related to the broader ability to read and interpret social behavior in everyday interactions, with all of its cultural display rules. As children learn that someone can act in a realistic manner but not experience what they are portraying, they may be able to apply this to understanding how social rules can affect behavior but not internal experience.

In the same way we are asking children to distinguish “good” acting it may be interesting to see whether and how children distinguish “good” pretense, which is behaviorally cued to its representational nature. As far as we know, no research has investigated whether children believe someone who is pretending in an announced, exaggerated way is a “better” pretender than someone who is pretending in an understated way. Children may believe that the more exaggerated the behaviors and less representational the actions and props of the pretense, the “better” children consider it to be. Future work will distinguish between the framing of a behavior (i.e. whether it is called pretend or acting) and the behavioral cues themselves (i.e. whether they are realistic or non realistic in nature) to determine children’s preferences given contrasts between the labels and cues.

Finally, how do children then come to understand the realistic representations of acting? We see three non-exclusive possibilities.

One possibility is while children are first exposed to the nonrealistic enacted world of children’s television, as they grow, the type of television and therefore the type of acting they are exposed to changes as well. This predicts that as children begin to watch more realistically acted television programs, their understanding of realistic acting will also develop. In particular, it may be that children’s understanding of acting (in our studies) is moderated by their understanding television as unreal but representational. Beginning at age 3, children begin to learn that objects on television are not actual objects existing within the square box of the screen, and by age four, children realize that the objects seen on the screen are representations [34]. At four, children also begin to understand that the world they see on television is separate from their own and cannot be changed [35]. Some work has shown that preschool children understand that cartoons are not real [36], but other work has shown until age 5, children do not understand the status of cartoon and live action televisions. At age 5, children begin to understand that television is pretend, but still believe that the characters that they see could be real and retain their jobs after the end of a television program [29]. In general the presence of live actors is the factor that causes the most confusion for preschool children’s understanding of TV’s reality status [37]. It may be that as children’s understanding of television genres and reality status becomes more adult-like, so do their judgments of the acting they see on television.

A second related possibility is that given the complex layering of representations in acting (i.e. the actor, the character, and the story), as representational abilities increase with age, so does understanding of acting and judgments of what makes an actor good at the task of acting. As children become better able to distinguish the appearance of realistic behavioral cues from the reality of a fictional portrayal, they will be more adult-like in their judgments of acting. Children will then understand, as adults do, the activity of realistically portraying the personality, emotions and physical states of a fictional being onstage or onscreen.

Correspondingly, although no work that we know of has explicitly investigated the acting abilities of children, previous research has shown that while children can engage in pretense easily, with the associated cues to its falsity, preschool aged children have difficulties convincingly lying—that is, they cannot perceptually act as though the lie is reality while the truth of the situation is something different [38]. Child actors are also rare (the old W.C. Fields saying is “never work with children or animals”), owing perhaps to the fact that getting young children to act realistically is very difficult. No child has ever won an Oscar for best Actor, or Actress, and only three children under the age of 14 (two nine-year olds and one 13 year old) have ever been nominated. For Supporting Actor or Actress, the youngest winner is Tatum O’Neal at 10 years, 5 months old, far older than our participants. No one under the age of 8 has ever been nominated [39].

A third possibility is that as children’s other social cognitive abilities, such as the ability to separate fiction from reality, appearance from reality, and have a complex theory of mind continue to develop, so does their ability to understand the fictional but realistic nature of characteristic portrayals in acting. If this is true, understanding acting may be linked to understanding the social world and mental and emotional states in general. Future work in our laboratory will explore these possibilities.

Although realistic acting is pervasive in modern culture, children do not understand how it works. Even with the obvious quarantining of a television set, children have problems understanding acting. This lack of understanding lags behind how well children reason about the fiction/ reality barrier and pretense in general. We propose that acting’s multiple layers make it a unique case study into children’s developing metarepresentational understanding and deserving of further exploration.
